# Supplementary material for: Identification, Analysis and Gene Cloning of the SWEET Gene Family Provide Insights into Sugar Transport in Pomegranate (Punica granatum)
Source: Int J Mol Sci. 2022 Feb 23;23(5):2471. doi: 10.3390/ijms23052471 (PMC8909982; doi:10.3390/ijms23052471)
Supplement: Supplementary file 1 [file ijms-23-02471-s001.zip › ijms-1597357-supplementary.pdf]

Table S1. Primers for the gene qRT-PCR.

Primer	Primer sequence（5′-3′）
PgSWEET1c	F：GTTTCCCTCCTTGCGTTG<br>R：GGCATATACTCGACGCTCT
PgSWEET1e	F：CTTCCTGATATTCGCTCCC<br>R：CCGCACAAGAACACGAAC
PgSWEET2	F：TGCCATACATTTACGCCCTC<br>R：AGCGAAGCCACTTATCACC
PgSWEET3	F：AACTGCCTCCTCTACACC<br>R：GCATGAACTCAACGCTCT
PgSWEET5	F：TCCGCAGAAAGATCCTCGT<br>R：AGGGCTAACATTGTCACCA
PgSWEET7a	F：CTAACAGCACCCTCGTCA<br>R：ATCCCGACGATCATGGAC
PgSWEET7b	F：CTAACAGCACCCTCGTCA<br>R：ATCCCGACGATCATGGAC
PgSWEET9	F：GCCGTTCACTCTGTCGTT<br>R：TCGTCTCTTCGTGTTGCT
PgSWEET10	F：CATTCACCAGCCATCGTC<br>R：CGTCAGCATCCTAGCCTT
PgSWEET11	F：GCCTTCTTTTCCGTCATCGT<br>R：CAATCCCGAATGCAAAGCC
PgSWEET12	F：CCTCCTGCTGAACATTGGT<br>R：CGAGCCATATCACCGCATT
PgSWEET15	F：AGGCAAGTTCTCAAGACCA<br>R：AGAGACCATAGCCAAACCAC
PgSWEET16a	F：CTTCCTCATATACGCTCCCA<br>R：AGCAACGCATAAGTAGACCA
PgSWEET16b	F：ATCCCCATACATAACAACCC<br>R：AGTATGCCGTAGAATGTCCA
PgSWEET17a	F：GCCGTTCTTCCTCTCGTT<br>R：TTTTGTTCCCTGCGATGGCT
PgSWEET17b	F：ATTCCAAATGTGACCGGACT<br>R：AGCTGAAATATCCCCAAGCC
